# Effect of Methotrexate on Semen Parameters in Psoriatic Male Patients

**DOI:** 10.7759/cureus.85787

**Published:** 2025-06-11

**Authors:** Samir Elhanbly, Doaa Shendy, Marwa Zohdy

**Affiliations:** 1 Dermatology, Andrology and Sexually Transmitted Diseases, Faculty of Medicine, Mansoura University, Mansoura, EGY

**Keywords:** male fertility, methotrexate, mtx safety, psoriasis, semen parameters

## Abstract

Background

Psoriasis is a chronic, immune-mediated inflammatory skin disease that affects approximately 60 million people worldwide. Methotrexate (MTX), a folate antagonist, is frequently prescribed as a systemic treatment for moderate-to-severe psoriasis due to its immunosuppressive efficacy and cost-effectiveness. Despite its long-standing use, concerns persist regarding its potential impact on male fertility, particularly on semen parameters.

Objective

To evaluate the impact of methotrexate treatment on semen parameters in male patients with psoriasis.

Methods

This prospective pre-post cohort study included 31 male psoriasis patients aged 18-50, treated with MTX for at least three months. Patients with systemic illnesses (e.g., diabetes, cardiovascular disease), endocrine disorders (e.g., thyroid dysfunction), autoimmune diseases (e.g., lupus, rheumatoid arthritis), genetic syndromes, or recent hormonal therapy (e.g., testosterone, anabolic steroids, or gonadotropins) within three months were excluded. Semen analyses were conducted immediately before initiating MTX therapy and again after completing three continuous months of treatment, without discontinuation, Data were analyzed using a paired t-test to compare pre- and post-treatment continuous variables, including semen volume, pH, sperm concentration, motility, morphology, and pus cell count (WBCs) using WHO 2010 criteria. Categorical data were summarized using frequencies and percentages. A p-value < 0.05 was considered statistically significant.

Results

The mean age of the study participants was 34.84 ± 9.78 years. No statistically significant differences (all p > 0.05) were observed pre- and post-treatment in semen volume (3.01 ± 1.61 vs. 3.12 ± 0.88 mL), pH (7.57 ± 0.11 vs. 7.56 ± 0.096), total sperm motility (46.61 ± 20.97% vs. 46.01 ± 21.14%), sperm morphology (19.26 ± 9.99% vs. 19.49 ± 11.90%), sperm concentration (50.21 ± 21.75 vs. 61.20 ± 29.56 million/mL), or pus cell count in semen (2.94 ± 2.29 vs. 2.80 ± 2.28).

Conclusion

Methotrexate treatment over three months did not lead to statistically significant changes in semen parameters among psoriatic male patients, indicating no observed short-term adverse effects on male reproductive indicators. Given the small sample size and absence of a control group, these findings should be considered preliminary and warrant further validation in larger, controlled studies.

## Introduction

Psoriasis is a chronic, immune-mediated, multidomain disease involving the skin, joints, and other systemic components. It is associated with various coexisting conditions, including psoriatic arthritis, psychiatric disorders such as depression and anxiety, cardiovascular diseases (e.g., coronary artery disease, hypertension), and hepatic dysfunction (e.g., non-alcoholic fatty liver disease) [1]. The most common clinical subtype is plaque psoriasis, characterized by well-demarcated, salmon-pink plaques covered with silvery-white scales. Lesions typically appear symmetrically on extensor surfaces, especially the elbows, knees, trunk, and scalp. Bleeding points may occur upon removal of scales, a feature known as the Auspitz sign.

Psoriasis affects both males and females and often manifests earlier in females and individuals with a family history. Globally, it affects an estimated 60 million individuals, with prevalence ranging from 0.05% in Taiwan to 1.88% in Australia. Higher prevalence is observed in high-income countries; for example, in the UK, psoriasis affects approximately 1.52% of the general population and is more common among aging individuals [1].

The pathogenesis of psoriasis is multifactorial, with genetics being a primary contributor, particularly in individuals with early-onset plaque psoriasis. This is supported by twin studies, family-based research, and large-scale population analyses, which estimate heritability between 60% and 90%. Numerous susceptibility genes have been linked to psoriasis pathogenesis, including those involved in antigen processing (e.g., *HLA-C*, *ERAP1*), NF-κB signaling (e.g., *TNIP1*), type I interferon response (e.g., *RNF113*, *IFIH1*), IL-23/Th17 immune axis (e.g., *IL23R*, *IL12B*, *TYK2*), and skin barrier maintenance (e.g., *LCE3*). These genetic associations point to a multifaceted immune-mediated mechanism, where dysregulation of T cells, dendritic cells, and keratinocytes, particularly through IL-23/Th17 signaling, plays a central role in driving inflammation, immune activation, and keratinocyte hyperproliferation. Environmental triggers have also been recognized in exacerbating the disease, including obesity, stress, beta-blocker use, smoking, and lithium exposure [1,2].

Management includes topical agents, phototherapy, and systemic treatments. First-line topical therapies include corticosteroids or vitamin D analogues (e.g., calcipotriol), often used under occlusion or in combination regimens [1]. Second-line treatments include phototherapy (e.g., narrowband UVB (NB-UVB), Psoralen ultraviolet light A (PUVA)) and systemic agents such as methotrexate, ciclosporin, and acitretin [1,3]. Methotrexate remains one of the most commonly prescribed systemic treatments for moderate-to-severe psoriasis and psoriatic arthritis worldwide. It is frequently used as a first-line systemic agent and serves as the backbone of many combination regimens, including those involving biologics. Methotrexate is effective in addressing both cutaneous manifestations and joint involvement, including enthesitis and dactylitis [4-6]. As a conventional disease-modifying antirheumatic drug (cDMARD), methotrexate is typically administered orally or parenterally (subcutaneous or intramuscular) and initiated at an average dose of 10-15 mg per week. However, the optimal starting dose should be individualized based on disease severity and patient factors [7].

However, despite the long-standing use of methotrexate in the treatment of immune-mediated inflammatory conditions, its impact on male reproductive health remains insufficiently explored. Concerns persist regarding potential effects on semen quality and fertility outcomes, particularly in younger male patients. A recent meta-analysis and prospective cohort study by Perez-Garcia et al. concluded that low-dose methotrexate did not adversely affect semen parameters or fertility outcomes in men with inflammatory diseases, yet it also emphasized the limited availability of high-quality, controlled studies addressing this issue [8]. This highlights an important knowledge gap, especially in the context of psoriasis. Therefore, the present study was conducted to evaluate the potential effects of methotrexate therapy on semen parameters in male patients with psoriasis.

## Materials and methods

Study design and setting

This prospective pre-post cohort study included 31 male patients diagnosed with psoriasis who were prescribed methotrexate (MTX) monotherapy for three months. Patients were recruited from the Dermatology and Andrology Outpatient Clinic at the Department of Dermatology, Andrology, and STDs, Mansoura University Hospital, between June 2023 and June 2024. The study was designed to evaluate intra-individual changes in semen parameters before and after MTX exposure, without the inclusion of a control group. This study was exploratory in nature and aimed to generate preliminary clinical insights into the reproductive impact of MTX in psoriatic males. A formal power calculation was not performed due to the absence of sufficient prior data on semen outcomes in this patient population.

Inclusion criteria

Eligible participants were male patients aged 18 to 50 years, diagnosed with psoriasis, for whom MTX monotherapy was initiated without concurrent systemic, topical, or phototherapy. Participants were required to have completed a continuous MTX course of at least three months. A three-month treatment duration was selected to align with the average length of one full spermatogenesis cycle (~74 days), thereby enabling the detection of treatment-related changes in semen quality.

Exclusion criteria

Patients were excluded if they had systemic (e.g., diabetes, cardiovascular disease), endocrine (e.g., thyroid dysfunction), autoimmune (e.g., lupus, rheumatoid arthritis), or genetic disorders; if they had received hormonal therapy (e.g., testosterone, gonadotropins, or anabolic steroids) within the past three months; or had a known history of testicular failure, including primary causes (e.g., Klinefelter syndrome) or secondary causes (e.g., pituitary insufficiency). Patients with andrological conditions such as varicocele, orchitis, or prostatitis were also excluded.

Patients on combination systemic therapies, other immunosuppressants, or biologics were excluded. Individuals with active infections, including tuberculosis (TB) or human immunodeficiency virus (HIV), or those undergoing other concurrent immunosuppressive therapy, were also excluded, given the potential immunomodulatory effects on fertility and ethical constraints related to immunocompromised populations.

Ethical considerations

The study was approved by the Institutional Review Board (IRB) of the Faculty of Medicine, Mansoura University (Approval No. MS.23.05.2398-2023/5/28). Written informed consent was obtained from all participants following a detailed explanation of the study objectives and procedures. Participation was voluntary, and patients were free to withdraw at any time without consequence. Confidentiality was ensured, and all collected data were used solely for research purposes.

Clinical assessment

Each participant underwent comprehensive history taking, general physical examination, and full dermatological evaluation of the skin, scalp, nails, and mucous membranes. This assessment determined the clinical type, distribution, and severity of psoriasis and excluded autoimmune dermatoses. Disease severity was evaluated using the Psoriasis Area and Severity Index (PASI), which scores erythema, scaling, and lesion thickness, adjusted for body surface area involvement [9]. The mean PASI score of participants prior to methotrexate treatment was 16, with a range of 10 to 25.

Semen analysis

Semen samples were obtained immediately before initiating MTX therapy and after three months of treatment. Samples were collected by masturbation into sterile containers following a period of sexual abstinence of 2 to 7 days. ‏Samples were left to liquefy at 37°C and were analyzed just after liquefaction (within an hour) using Computer-Assisted Semen Analysis *(*CASA; Mira-9000 system; Mira Lab, Cairo, Egypt*)*. Parameters assessed included semen volume (mL), sperm concentration (million/mL), total motility (%), normal morphology (%), pH, and pus cell count (WBCs). All assessments were conducted in accordance with the World Health Organization (WHO) 2010 guidelines [10].

Treatment protocol and follow-up

Methotrexate was administered for a duration of three months. Oral doses ranged from 7.5 to 25 mg once weekly, with an average dose of 15 mg/week across participants. Parenteral administration, delivered via subcutaneous, intramuscular, or intravenous routes, ranged from 2.5 to 25 mg weekly depending on individual clinical needs. Dose titration was guided by disease severity and tolerability, with escalation or reduction as clinically indicated. Folic acid supplementation (5 mg/week) was provided routinely to all participants during treatment. After the three-month treatment course, a follow-up visit was conducted to reassess semen parameters and evaluate any changes resulting from methotrexate therapy.

Statistical analysis

Continuous variables were presented as means ± standard deviations (SD). Pre- and post-treatment comparisons were conducted using paired t-tests. No statistical tests were applied to categorical classifications of semen volume. The normality of paired differences was assessed using the Shapiro-Wilk test, confirming the appropriateness of parametric testing. Categorical variables were expressed as frequencies and percentages. A p-value < 0.05 was considered statistically significant.

## Results

The number of study participants was 31 psoriatic male patients. The mean age of the study participants was 34.84 ± 9.78 years. To provide descriptive insight into the sample distribution, age was stratified at 40 years, as this represents a relevant threshold in fertility-related literature due to the reported age-related decline in semen quality. The majority, 74.2% (n = 23) were aged 40 years or younger, while 25.8% (n = 8) were older than 40 years. In terms of residence, 54.8% (n = 17) lived in urban areas and 45.2% (n = 14) in rural areas. Smoking was reported by 51.6% (n = 16) of the patients (Table 1).

**Table 1:** Sociodemographic characteristics of the study participants Data are presented as absolute numbers (n) and relative frequencies (%). No statistical testing was performed, as these are descriptive characteristics.

Variable	Category	n (%)
Age (years)	≤ 40	23 (74.2%)
	> 40	8 (25.8%)
Residence	Urban	17 (54.8%)
	Rural	14 (45.2%)
Smoking Status	Non-smoker	15 (48.4%)
	Smoker	16 (51.6%)

The mean semen volume increased slightly from 3.01 ± 1.61 mL pre-treatment to 3.12 ± 0.88 mL post-treatment. However, this difference was not statistically significant (t = 0.386, p = 0.702), with a mean difference of 0.116 (95% CI: -0.300 to 1.67). The relative percentage change observed was 3.7%. Additionally, the proportion of patients with abnormal semen volume (hypospermia or hyperspermia) decreased from 19.4% (n = 6) to 9.7% (n = 3) following treatment (Table 2).

**Table 2:** Comparison of semen volume before and after methotrexate treatment CI: confidence interval. Data are presented as mean ± standard deviation (SD), absolute numbers (n) and relative frequencies (%). Statistical comparisons of pre- and post-treatment values were performed using a paired t-test for continuous variables. No statistical tests were applied to categorical classifications of semen volume. Analysis was based on 31 paired observations. A p-value < 0.05 was considered statistically significant. Percentage change refers to the relative change in mean values between pre- and post-treatment measurements.

Parameter	Pre-treatment	Post-treatment	t-Value	p-value	Mean Difference (95% CI)	% Change
Semen Volume (mL)	3.01 ± 1.61	3.12 ± 0.88	0.386	0.702	0.116 (−0.300 to 1.67)	3.7%
Abnormal Volume, n (%)	6 (19.4%)	3 (9.7%)	—	—	—	—
Normal Volume, n (%)	25 (80.6%)	28 (90.3%)	—	—	—	—

Semen pH was analyzed to determine whether methotrexate administration influenced the acid-base balance of seminal fluid. The mean pH showed a minimal, non-significant change from 7.57 ± 0.11 to 7.56 ± 0.096 after treatment (t = 1.04, p = 0.305), with a mean difference of 0.016 (95% CI: −0.015 to 0.047) and a percentage change of 0.21% (Table 3). Notably, all participants (100%, n = 31) maintained semen pH values within the normal reference range throughout the study.

**Table 3:** Comparison of semen pH before and after methotrexate treatment CI: confidence interval. Data are presented as mean ± standard deviation (SD), absolute numbers (n) and relative frequencies (%). Statistical comparisons of pre- and post-treatment values were performed using a paired t-test for continuous variables. No statistical comparisons were performed for categorical data where values remained constant across groups. Analysis was based on 31 paired observations. A p-value < 0.05 was considered statistically significant. Percentage change refers to the relative change in mean values between pre- and post-treatment measurements.

Parameter	Pre-treatment	Post-treatment	t-value	p-value	Mean Difference (95% CI)	% Change
Semen pH	7.57 ± 0.11	7.56 ± 0.096	1.04	0.305	0.016 (−0.015 to 0.047)	0.21%
Abnormal pH, n (%)	0 (0%)	0 (0%)	—	—	—	—
Normal pH, n (%)	31 (100%)	31 (100%)	—	—	—	—

Total sperm motility was assessed to examine any methotrexate-related changes in sperm movement efficiency (Table 4). The mean motility slightly decreased from 46.61 ± 20.97% before treatment to 46.01 ± 21.14% after treatment; however, this change was not statistically significant (t = 0.152, p = 0.880). The mean difference was 0.584 (95% CI: −7.28 to 8.45), indicating a 1.3% change. The proportion of patients with abnormal motility increased from 35.5% (n = 11) to 41.9% (n = 13), while the percentage with normal motility decreased from 64.5% (n = 20) to 58.1% (n = 18).

**Table 4:** Comparison of total sperm motility before and after methotrexate treatment CI: confidence interval. Data are presented as mean ± standard deviation (SD), absolute numbers (n) and relative frequencies (%). Statistical comparisons of pre- and post-treatment values were performed using a paired t-test for continuous variables. No statistical comparisons were performed for categorical classifications of motility status. Analysis included 31 paired observations. A p-value < 0.05 was considered statistically significant. Percentage change refers to the relative change in mean values between pre- and post-treatment measurements.

Parameter	Pre-treatment	Post-treatment	t-value	p-value	Mean Difference (95% CI)	% Change
Total Sperm Motility (%)	46.61 ± 20.97	46.01 ± 21.14	0.152	0.880	0.584 (−7.28 to 8.45)	1.3%
Abnormal Motility, n (%)	11 (35.5%)	13 (41.9%)	—	—	—	—
Normal Motility, n (%)	20 (64.5%)	18 (58.1%)	—	—	—	—

Sperm concentration was evaluated to assess potential changes in sperm density following methotrexate therapy. The mean concentration increased from 50.21 ± 21.75 ×10⁶/mL prior to treatment to 61.20 ± 29.56 ×10⁶/mL afterward (Table 5). Although this upward trend approached significance, it did not reach the statistical threshold (t = 1.86, p = 0.051). The mean difference was −10.99 (95% CI: −21.50 to −0.487), reflecting a 21.9% increase. Importantly, the proportion of patients with abnormal sperm concentration declined from 3.2% (n = 1) to 0.0% (n = 0). Given the p-value and limited sample size, this trend should be interpreted with caution and considered exploratory rather than confirmatory.

**Table 5:** Comparison of sperm concentration before and after methotrexate treatment CI: confidence interval. Data are presented as mean ± standard deviation (SD), absolute numbers (n) and relative frequencies (%). Statistical comparisons of pre- and post-treatment values were performed using a paired t-test for continuous variables. No statistical testing was applied to categorical classifications of sperm concentration. Analysis included 31 paired observations. A p-value < 0.05 was considered statistically significant. Percentage change refers to the relative change in mean values between pre- and post-treatment measurements.

Parameter	Pre-treatment	Post-treatment	t-value	p-value	Mean Difference (95% CI)	% Change
Sperm Concentration (×10⁶/mL)	50.21 ± 21.75	61.20 ± 29.56	1.86	0.051	−10.99 (−21.50 to −0.487)	21.9%
Abnormal Concentration, n (%)	1 (3.2%)	0 (0.0%)	—	—	—	—
Normal Concentration, n (%)	30 (96.8%)	31 (100.0%)	—	—	—	—

Sperm morphology was assessed to evaluate any structural changes in spermatozoa following methotrexate administration (Table 6). The mean percentage of morphologically normal sperm increased slightly from 19.26 ± 9.99% before treatment to 19.49 ± 11.90% post-treatment. This difference was not statistically significant (t = 0.145, p = 0.866), with a mean difference of −0.24 (95% CI: −3.64 to 1.66), corresponding to a 1.2% change. All participants (100%, n = 31) maintained sperm morphology values within the normal reference range throughout the study.

**Table 6:** Comparison of sperm morphology before and after methotrexate treatment CI: confidence interval. Data are presented as mean ± standard deviation (SD), absolute numbers (n) and relative frequencies (%). Statistical comparisons of pre- and post-treatment values were performed using a paired t-test for continuous variables. No statistical testing was applied to categorical data where all values were constant. Analysis included 31 paired observations. A p-value < 0.05 was considered statistically significant. Percentage change refers to the relative change in mean values between pre- and post-treatment measurements.

Parameter	Pre-treatment	Post-treatment	t-value	p-value	Mean Difference (95% CI)	% Change
Morphologically normal sperm (%)	19.26 ± 9.99	19.49 ± 11.90	0.145	0.866	−0.24 (−3.64 to 1.66)	1.2%
Below Normal Range, n (%)	0 (0%)	0 (0%)	—	—	—	—
Within Normal Range, n (%)	31 (100%)	31 (100%)	—	—	—	—

Pus cell count in semen was measured as an indicator of potential subclinical inflammation or infection (Table 7). Following methotrexate treatment, the mean count showed a slight, non-significant decrease from 2.94 ± 2.29 to 2.80 ± 2.28 (t = 0.282, p = 0.780). The mean difference was 0.142 (95% CI: −0.885 to 1.17), corresponding to a 4.8% reduction. Notably, the proportion of patients with abnormal pus cell counts increased slightly from 93.5% (n = 29) to 96.8% (n = 30) post-treatment.

**Table 7:** Comparison of pus cell count before and after methotrexate treatment CI: confidence interval. Data are presented as mean ± standard deviation (SD), absolute numbers (n) and relative frequencies (%). Statistical comparisons of pre- and post-treatment values were performed using a paired t-test for continuous variables. No statistical analysis was applied to categorical data classifications. Analysis was based on 31 paired observations. A p-value < 0.05 was considered statistically significant. Percentage change refers to the relative change in mean values between pre- and post-treatment measurements.

Parameter	Pre-treatment	Post-treatment	t-value	p-value	Mean Difference (95% CI)	% Change
Pus Cell Count	2.94 ± 2.29	2.80 ± 2.28	0.282	0.780	0.142 (−0.885 to 1.17)	4.8%
Abnormal Count, n (%)	29 (93.5%)	30 (96.8%)	—	—	—	—
Normal Count, n (%)	2 (6.5%)	1 (3.2%)	—	—	—	—

## Discussion

Methotrexate remains the most widely prescribed systemic therapy for moderate-to-severe psoriasis. Given its extensive use, evaluating its potential impact on male reproductive health is of clinical importance. In this context, the present study employed a pre-post cohort design to assess changes in semen parameters among male psoriatic patients over a three-month methotrexate treatment period. The mean age of participants was 34.84 ± 9.78 years, with the majority (74.2%, n = 23) aged ≤40 years, and 25.8% (n = 8) older than 40 years. Participants were nearly evenly distributed by residence, with 54.8% (n = 17) residing in urban areas and 45.2% (n = 14) in rural areas. Additionally, 51.6% (n = 16) of patients reported current smoking.

Solmaz et al. reported that 444 (31.9%) of 1,393 psoriasis patients had a family history of psoriasis and/or psoriatic arthritis [11]. Similarly, Jiang et al., in a larger cohort of 5,961 patients with psoriasis, found a family history in 912 (15.3%) of patients, with 5,071 (85.1%) presenting with plaque psoriasis [12]. In terms of semen analysis, no statistically significant difference was found in semen volume following treatment (3.01 ± 1.61 mL vs. 3.12 ± 0.88 mL; p > 0.05), with a modest percentage increase of 3.7%. These results align with findings by Pérez-García et al., who observed no significant differences in semen volume, sperm concentration, or motility between methotrexate-naïve patients (i.e., had not received prior methotrexate treatment) and healthy controls, as well as in chronic methotrexate users [8]. Notably, two episodes of oligospermia (<15 × 10⁶/mL) were reported in a single naïve patient, but no cases of azoospermia were detected. No significant changes were detected in semen pH (7.57 ± 0.11 vs. 7.56 ± 0.096; p > 0.05; 0.21% change) or total sperm motility (46.61 ± 20.97% vs. 46.01 ± 21.14%; p > 0.05; 1.3% change) after treatment. Pérez-García et al. similarly reported stable semen quality, with only one case of oligospermia among treated individuals [8]. El Behery et al. investigated 26 psoriasis patients and found no significant post-treatment alterations in sperm count, morphology, or motility after 70 days of oral methotrexate at 25 mg/week [13]. Although sperm analysis during treatment was not conducted, histological assessments via testicular biopsies in five patients indicated unaffected spermatogenic activity.

In the present study, sperm concentration showed an upward trend (50.21 ± 21.75 ×10⁶/mL vs. 61.20 ± 29.56 ×10⁶/mL), but the difference did not reach statistical significance (p > 0.05), despite a 21.9% increase. Sperm normal morphology also remained largely unchanged (19.26 ± 9.99% vs. 19.49 ± 11.90%; 1.2% change). Likewise, pus cell count demonstrated a slight, non-significant reduction (2.94 ± 2.29 vs. 2.80 ± 2.28; 4.8% change), although the proportion of patients with abnormal counts slightly increased from 93.5% (n = 29) to 96.8% (n = 30) post-treatment. In contrast to these findings, Van Scott and Reinertson reported a marked reduction in sperm count (spermatogenesis) two weeks following a single intravenous methotrexate dose in two psoriasis patients [14]. This discrepancy suggests that administration route and dosage intensity may play a critical role in influencing reproductive outcomes.

Study limitations

The relatively small sample size (n = 31), absence of a control group, and the short follow-up duration of three months limit the generalizability of the results. Additionally, hormonal profiling and long-term fertility outcomes, such as conception rates or pregnancy outcomes, were not assessed. Semen functional assays, including DNA fragmentation index or oxidative stress markers, were also not performed, which could provide deeper insights into subclinical reproductive changes. Future research should incorporate these parameters and consider multicenter collaboration to improve statistical power, patient diversity, and broader external validity. Larger, controlled cohorts with extended follow-up and comprehensive endocrine evaluation are essential to confirm and expand upon these preliminary findings.

## Conclusions

In the management of psoriasis, methotrexate appears to be a relatively safe systemic therapeutic option with no statistically significant adverse effects on semen quality among male patients of reproductive age. The findings of this study support the consideration of methotrexate as a viable treatment for men with psoriasis who are planning for fatherhood. With appropriate pre-treatment counseling and routine reproductive health monitoring, methotrexate may be prescribed without substantial concerns regarding its impact on male fertility.
